# Prophylactic closed-incisional negative pressure wound therapy following posterior instrumented spinal fusion: a single surgeon’s experience and cost-benefit analysis

**DOI:** 10.1007/s10143-024-03083-8

**Published:** 2024-11-15

**Authors:** Dallas E. Kramer, Rosh Bharthi, Daniel Myers, Patrick Chang, Rocco Dabecco, Chen Xu, Alexander Yu

**Affiliations:** 1 Department of Neurosurgery, Allegheny Health Network, Pittsburgh, PA 15212 USA; 2 Lake Erie College of Osteopathic Medicine, Erie, PA 16509 USA; 3 Drexel University College of Medicine, Philadelphia, PA 19129 USA

**Keywords:** Cost-benefit analysis, Negative pressure wound therapy, Spinal fusion, Surgical site infection, Vacuum-assisted closure

## Abstract

Purpose: Surgical site infections (SSIs) following spine surgery are associated with morbidity and resource utilization. Applying prophylactic closed-incisional negative pressure wound therapy (ciNPWT) during posterior instrumented fusion has mixed results in reducing rates of wound complications and SSI. We evaluated the clinical efficacy and potential cost-savings associated with ciNPWT in high-risk patients receiving posterior instrumented spinal fusion. Methods: We retrospectively reviewed patients receiving posterior instrumented spinal fusion for any surgical indication between July 1, 2017 and December 31, 2019, and compared rates of wound dehiscence, SSI, and reoperation for wound complications between standard surgical dressings and ciNPWT. Surgical dressing selection was based on the senior author’s assessment of infection risk factors. Results: A total of 229 patients (*n* = 85 standard surgical dressings, *n* = 144 ciNPWT) were included. The ciNPWT group had significantly more risk factors for wound-related complications, including older age (61.8 vs. 58.5 years, *p* = 0.042), diabetes mellitus (36.8% vs. 23.5%, *p* = 0.037), more instrumented levels (5.6 vs. 3.9, *p* < 0.0001), estimated blood loss (1298 vs. 998 mL, *p* = 0.036), and deformity was the predominant operative indication (29.9% vs. 17.7%, *p* = 0.040). Prophylactic ciNPWT was associated with significantly lower rates of wound dehiscence (21.5% vs. 34.1%, *p* = 0.036) and SSI (8.3% vs. 21.2%, *p* = 0.005). Number needed to treat with ciNPWT to prevent one SSI was 8 patients. The cost of preventing one SSI was $4,560. Cost-benefit analysis demonstrated a potential mean savings of $21,662 per operative SSI prevented and $270,775 per 100 patients undergoing posterior instrumented fusion with ciNPWT. Conclusions: Prophylactic ciNPWT use is a cost-effective means of reducing rates of wound dehiscence and SSI following posterior instrumented fusion.

**Supplementary Information:**

The online version contains supplementary material available at 10.1007/s10143-024-03083-8.

## Previous presentations

An abstract from this work was presented as an oral presentation at the 2023 CNS Annual Meeting in Washington, DC.

**Supplementary Information:**

The online version contains supplementary material available at 10.1007/s10143-024-03083-8.

## Introduction

Surgical site infections (SSIs) and wound dehiscence are sources of significant morbidity, reoperation, and increased healthcare-associated costs following instrumented spine surgery [1–3]. Total direct costs associated with treatment of postoperative spine SSI range from $16,242 to $31,245 [3, 4] Identified risk factors for SSI following spinal surgery include increased age, male sex, history of coronary artery disease, hypertension, diabetes mellitus, obesity (body mass index [BMI] > 30 kg/m^2^), chronic steroid use, higher American Society of Anesthesiologists (ASA) classification, *≥* 3 h operative time, and transfusion, among others [5–9]. As such, preventative strategies to reduce the incidence of SSI have garnered increasing importance. Closed-incisional negative pressure wound therapy (ciNPWT) is one such strategy, which is thought to facilitate wound healing through wound approximation, wound environment stabilization, promotion of angiogenesis, granulation tissue formation, and a decreased bacterial burden [10]. In contrast to the application of negative pressure wound therapy (NPWT) for healing by secondary intention, whereby the dressing is applied within an open incision, a ciNPWT involves a wound VAC dressing applied over the closed incision.

A 2019 proof-of-concept study which selected patients with increased risk of SSI to receive ciNPWT, found that closed-incisional vacuum-assisted closure (VAC) application resulted in a 50% reduction in the rate of SSI relative to standard dressing [11]. Additional retrospective and prospective studies which have suggested potential benefit of ciNPWT in reducing rates of wound dehiscence and SSI [4, 12–15]. Pooled analysis showed a significantly lower incidence of SSI with application of ciNPWT, but no significant reduction in the incidence of wound dehiscence, wound complication, or reoperation [16]. While ciNPWT is initially more costly than other standard surgical dressings, a recent study limited to trauma patients reported a significant infection-related cost-savings with its use [4]. The objective of the present study was to assess the clinical efficacy and healthcare cost effectiveness of prophylactic ciNPWT use in patients undergoing posterior instrumented spinal fusion for any surgical indication.

## Materials and methods

### Study design and participants

This retrospective observational cohort study to assess the efficacy and cost effectiveness of ciNPWT was approved by the Institutional Review Board (IRB) and was exempted from patient consent by the IRB before initiation. All patients who underwent posterior instrumented spinal fusion performed by the senior author from July 2017 to December 2019 at a single Level 1 trauma center were reviewed. Starting in July 2017, the senior author developed a change of practice with the selective use of ciNPWT as a postoperative surgical dressing. Exclusion criteria included cases in which the patient was younger than 18 years of age and cases in which the senior author was not the primary surgeon.

Study cohort demographic and clinical variables included: age, sex, BMI, ambulatory status, ASA physical status classification, immunocompromised state (renal failure, liver failure, HIV, neutropenia [white blood cell count < 3.0], use of biological agents or chemotherapeutics), history of diabetes, tobacco use, chronic steroids, cancer, radiation to the operative location, indication for operation (trauma, degenerative, oncologic, infection), and whether the patient was receiving a revision surgery. Relevant operative details included: spine segment(s) involved, number of instrumented levels, operating time, estimated blood loss (EBL), and presence of intraoperative durotomy. Primary outcomes were incidence of postoperative wound dehiscence, SSI, and reoperation for wound complication.

### Surgical technique and postoperative care

All patients received weight-based intravenous antibiotics at the time of surgical incision with cefazolin (or vancomycin if the patient was allergic to penicillin) as well as perioperative antibiotics for 24 h. The surgical wound was irrigated with normal saline irrigation and subfascial closed-suction drains were placed. Standard wound closure was performed: muscle layers approximated with interrupted 0–0 vicryl, fascial layers tightly approximated with interrupted 0–0 vicryl and 2 − 0 vicryl, dermis approximated with inverted interrupted 2 − 0 vicryl, and skin approximated with staples.

Based on the surgeon’s assessment of the patient’s infection risk, after complete closure either a standard surgical dressing or closed-incisional wound VAC was used. Risk factors considered included but were not limited to history of diabetes or tobacco use, BMI (obesity/malnutrition), prolonged bedrest, anticipated postoperative radiation, and length of fusion. There was a low threshold to apply a ciNPWT for patients with more than one infection risk factor. In the control group, the skin was covered with a Primapore (Smith & Nephew, UK) adhesive wound dressing which were removed on postoperative day 2 after which the incision was left open to air. For the ciNPWT group, the Renasys Touch (Smith & Nephew, UK) system was used according to the manufacturer’s recommendation with negative pressure between 100 and 120 mmHg. The ciNPWT was continued until postoperative day 7 or discontinued at time of discharge from the hospital if sooner than postoperative day 7. After removal, the incision was left open to air. In both groups, closed-suction surgical drains were removed prior to discharge. Patients were instructed to follow-up on postoperative day 14 for staple removal.

If there were concerns regarding incisional healing at follow-up, staples were either left in place or the incision was oversewn at the site(s) of concern with 3 − 0 Nylon at the surgeon’s discretion. Patients were then instructed to follow-up 5–7 days later for re-evaluation. Indications for operative wound revision included wound dehiscence which did not resolve with conservative management and SSI. An SSI event was defined according to the Centers for Disease Control and Prevention (CDC) criteria: superficial SSI within 30 days or deep SSI within 30–90 days where implants are involved [17].

### Cost-benefit and statistical analysis

The number needed to treat (NNT) to prevent 1 SSI and cost of preventing an event were used to determine if an economic benefit for ciNPWT use existed. NNT was calculated as a reciprocal of the net effect of absolute risk reduction (ARR) with ciNPWT (NNT = 1 / ARR). Cost-savings per 100 patients receiving posterior instrumented spinal fusion treated with ciNPWT was calculated. Mean costs of ciNPWT and operative treatment of SSI were calculated from available health insurance claims data. Costs of ciNPWT therapy included the Primapore adhesive wound dressing and Renasys Touch system cost per day used. Costs of SSI treatment included costs all inpatient costs for the admission during which the reoperation occurred. Categorical variables were reported as frequency and percentages, and were analyzed using the chi-square test. Continuous variables were reported as mean *±* standard deviation, and were analyzed using the independent samples unpaired two-tailed student’s t-test. A *p* value < 0.05 was considered statistically significant.

## Results

### Patient population

A total of 229 patients were included for analysis. A ciNPWT was applied in 144 patients, while standard surgical dressings (control group) were applied in 85 patients. Baseline demographics are shown in Table 1. Patients treated with ciNPWT were significantly older (61.81 vs. 58.47 years, *p* = 0.042) and more likely to be diabetic (36.8% vs. 23.5%, *p* = 0.037) compared with controls.

**Table 1:** Demographics and clinical characteristics

	All Patients (%) *n* = 229	Control (%) *n* = 85	ciNPWT (%) *n* = 144	*p* value
Age	60.58 ± 12.10	58.47 ± 11.54	61.81 ± 12.21	***0.042***
Sex (Male)	116 (50.66%)	37 (43.53%)	79 (54.86%)	0.098
BMI	31.34 ± 8.35	30.67 ± 8.52	31.73 ± 8.25	0.355
Obese (BMI > 30)	123 (53.71%)	43 (50.59%)	80 (55.56%)	0.467
Malnourished (BMI < 18.5)	6 (2.62%)	2 (2.35%)	4 (2.78%)	0.846
Ambulatory Pre-op	195 (85.15%)	77 (90.56%)	118 (81.94%)	0.094
ASA Classification	2.92 ± 0.56	2.84 ± 0.69	2.97 ± 0.46	0.090
Immunocompromised	36 (15.72%)	15 (17.65%)	21 (14.58%)	0.538
Diabetes	73 (31.88%)	20 (23.53%)	53 (36.81%)	***0.037***
Tobacco Use	73 (31.88%)	27 (31.76%)	46 (31.94%)	0.977
Chronic Steroids	22 (9.61%)	10 (11.76%)	12 (8.33%)	0.394
History of Cancer	41 (17.90%)	11 (12.94%)	30 (20.83%)	0.132
Prior Radiation	14 (6.11%)	6 (7.06%)	8 (5.56%)	0.646

### Operative measures and patient outcomes

**Fig. 1:**
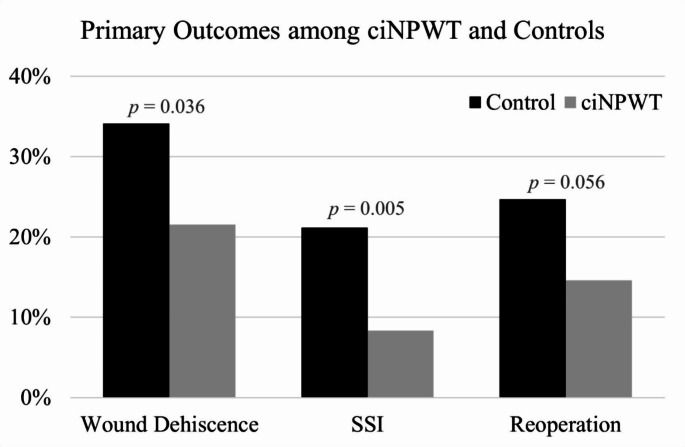
Graph showing a significant reduction in rates of wound dehiscence (*p* = 0.036) and SSI (*p* = 0.005) with incisional negative pressure wound therapy (iNPWT) use compared to standard surgical dressings. There was a trend towards a decreased rate of reoperation for wound complications with ciNPWT use as well (*p* = 0.056)

Degenerative disease was the most common indication among control patients and significantly more likely than ciNPWT patients (50.6% vs. 28.47%, *p* < 0.005). Meanwhile, deformity was the most common indication among ciNPWT patients and significantly more likely than controls (29.9% vs. 17.7%, *p* = 0.040). Incidences of operation for trauma, infection, and oncologic process were similar (Table 2). However, a significantly greater proportion of controls were operated on for degenerative disease (50.6% vs. 28.5%, *p* = 0.005). Patients within the ciNPWT group were treated with significantly more instrumented vertebral levels (5.60 *±* 2.74 vs. 3.87 *±* 2.58, *p* < 0.0001) than the control group. EBL was significantly greater among patients within the ciNPWT group (1298 vs. 998 mL, *p* = 0.005). Prophylactic ciNPWT use was associated with significantly lower rates of wound dehiscence (21.5% vs. 34.1%, *p* = 0.036). Most cases of wound dehiscence were managed non-operatively with continuation of staples and/or oversewing the wound with interrupted 3 − 0 Nylon. The incidence of reoperation for all wound-related complications was lower with ciNPWT (14.6% vs. 24.7%, *p* = 0.056), though it did not reach statistical significance. Incidence of SSI was significantly lower with ciNPWT (8.3% vs. 21.2%, *p* = 0.005) compared with standard surgical dressings (Fig. 1). Notably, all SSI in the present study received operative treatment and were readmitted to the hospital with a mean length of stay of 8 days.

**Table 2:** Operative measures and patient outcomes

	All Patients (*n* = 229)	Control (*n* = 85)	ciNPWT (*n* = 144)	*p* value
Follow-up (months)	17.71 ± 18.00	19.61 ± 19.49	16.77 ± 17.15	0.308
Operative Indication				
Trauma	34 (14.85%)	10 (11.76%)	24 (16.67%)	0.313
Degenerative	84 (36.68%)	43 (50.59%)	41 (28.47%)	***0.004***
Deformity	58 (25.32%)	15 (17.65%)	43 (29.86%)	***0.040***
Oncologic	27 (11.79%)	11 (12.94%)	16 (11.11%)	0.678
Infection	26 (11.35%)	6 (7.06%)	20 (13.89%)	0.115
Location				
Cervical	24 (10.48%)	9 (10.59%)	15 (10.42%)	0.967
Thoracic	29 (12.66%)	6 (4.17%)	23 (15.97%)	0.050
Lumbar	77 (33.62%)	44 (30.56%)	33 (22.92%)	***< 0.0001***
Cervicothoracic	48 (20.96%)	13 (9.03%)	35 (24.31%)	0.106
Thoracolumbar	50 (21.83%)	13 (9.03%)	37 (25.69%)	0.066
Cervicothoracolumbar	1 (0.44%)	0 (0.0%)	1 (0.69%)	0.441
Revision Surgery	53 (23.14%)	18 (21.69%)	35 (24.48%)	0.587
Instrumented Levels	4.96 ± 2.80	3.87 ± 2.58	5.60 ± 2.74	***< 0.0001***
Operative Time (min)	341.18 ± 163.62	330.82 ± 157.98	347.29 ± 167.10	0.463
EBL (mL)	1185.88 ± 1045.08	998.18 ± 1003.11	1298.24 ± 1056.97	***0.036***
Durotomy	13 (5.68%)	3 (3.53%)	10 (6.94%)	0.280
Wound Dehiscence	60 (26.20%)	29 (34.12%)	31 (21.53%)	***0.036***
Reoperation for Wound Complication	42 (18.34%)	21 (24.71%)	21 (14.58%)	0.056
Wound Dehiscence	12 (5.24%)	3 (3.53%)	9 (6.25%)	0.372
Surgical Site Infection	30 (13.10%)	18 (21.18%)	12 (8.33%)	***0.005***

### Cost-benefit analysis

Based on the differential incidence of SSIs, the calculated NNT with ciNPWT to prevent 1 SSI was 7.79. The relative risk reduction (RRR) was 60.65% and absolute risk reduction (ARR) was 12.84%. Average length of ciNPWT use was 6.63 *±* 4.34 days. Mean cost of ciNPWT use was $570, and the cost of SSI treatment was $26,222. The cost to prevent one SSI with ciNPWT was $4,560. Therefore, the potential mean cost savings associated with ciNPWT was $21,662 per SSI prevented and $270,775 per 100 patients undergoing posterior instrumented spinal fusion, irrespective of operative indication. The NNT to prevent 1 wound dehiscence was 7.94 (RRR 36.90%, ARR 12.59%) and NNT to prevent 1 reoperation for wound complication was 9.88 (RRR 40.97%, ARR 10.12%).

## Discussion

In the present study of patients receiving posterior instrumented spinal fusion, prophylactic ciNPWT use significantly decreased the rate of wound dehiscence by 36.9% and SSI by 60.7% compared with standard surgical dressings. This is despite the ciNPWT cohort being significantly biased towards increased risk factors for SSI, including older age, greater likelihood of diabetes, more instrumented levels, and EBL, and more operations for deformity correction [5, 7–9, 18].

Use of ciNPWT through VAC is an established means of reducing rates of SSI for surgical wound healing by primary closure [19]. Primary mechanisms of action attributed to the action of VAC devices used for ciNPWT include: (1) wound contraction (macrodeformation), (2) stabilization of wound environment, (3) removal of extracellular fluid, and (4) microdeformation at the foam-wound interface [10]. These primary changes yield the secondary changes desired by the surgeon, including enhanced angiogenesis and granulation tissue formation, favorable changes in wound biochemistry and the systemic inflammatory response, and a decrease in pathogen burden [10]. Individual reports have produced conflicting results regarding the efficacy of ciNPWT in reducing the incidence of SSI in spinal fusion surgery [4, 11, 13–15].

A prospective study among 274 patients by Mueller et al. supported the use of ciNPWT as a means of reducing rates of SSIs, especially among patients (instrumentation, deformity, and malignancy) and surgeries at higher risk, as well as patients with diabetes and higher EBL [15]. While there was no statistical difference in surgeries with decompression alone (4.2% vs. 9.1%, *p =* 0.63), there was a significant reduction in instrumented surgeries (3.4% vs. 10.9%, *p* = 0.02) [15]. A retrospective comparative study limited to spinal trauma patients by Mehkri et al. reported an independent association between ciNPWT use and decreased 90-day SSI and wound-related returns to the operating room [4]. This was despite a tendency towards ciNPWT patients possessing greater risk factors such as significantly older age, diabetes, and increased BMI. Cumulative 2022 meta-analysis reported that ciNPWT could significantly reduce the incidence of postoperative SSI, but there was no significant benefit on reducing the incidence of wound dehiscence or reoperation [13].

SSIs represent a significant cause of healthcare-acquired morbidity, mortality, and healthcare resource expenditure [1–3]. Annually in the United States, there are an estimated 158,000 SSIs at a cost of up to $10.07 billion in 2009 USD [20]. Spine surgeries account for more than 1 million procedures annually and neurosurgical SSIs incur the highest costs among surgical specialties, indicating an opportunity for significant harm and healthcare costs reduction [21]. Costs for treatment of postoperative spine surgery SSI range from $16,242 to $31,245 [3, 4]. In the present study, the NNT with ciNPWT to prevent one SSI was 8 patients. The incurred costs of using prophylactic ciNPWT ($4,560) to prevent one SSI requiring operative intervention ($26,222) amounts to a potential cost savings of $21,662 per SSI prevented. Per 100 patients receiving posterior instrumented spinal fusion, the potential mean cost-savings related to SSI prevention is $270,775. Mehkri et al. previously reported a potential cost-savings of $163,492 per 100 patients treated for spinal trauma [4]. The increased cost-savings in the present study may be attributed to inclusion of patients operated on for deformity, tumor, and infection, which carry a high risk of wound-related complications [14, 22–25]. Compared with Mehkri et al., patients in the present study possessed an even greater tendency towards risk factors including older age (60.58 vs. 52.42 years), increased BMI (31.34 vs. 28.44 kg/m^2^), cancer (17.9% vs. 2.9%), and chronic steroid use (9.6% vs. 0.5%) [4]. Increased age, including age > 60 years, has been shown to be significant preoperative risk factor [7–9]. These factors in addition to the inclusion of high-risk patients with infectious and oncologic surgical indications may account for the substantially greater cost-savings in the present study. Cost-benefit ratio in cases with lesser index surgery cost and risk of infection, such as decompression alone compared to instrument surgeries, may not warrant the higher surgical dressing costs. Further investigation should assist in developing an algorithm for the selective use of ciNPWT for increasingly complex cases (i.e. length of fusion, staged procedure) and higher-risk patient populations (neoplastic or infectious etiology) [14].

This is a retrospective study of a single surgeon’s experience among 229 patients that was not randomized and whose total cohort number resulted from an observational study and was not based on sample size calculation. Therefore, it is likely that our control group is underpowered to determine statistical significance of less frequently occurring demographic categories and clinical outcomes, including subgroup analysis of the effect of ciNPWT depending on surgical indication. The selection of patients to receive a standard surgical dressing versus ciNPWT was not based on a standardized algorithm and thus was subject to selection bias. However, patients in the ciNPWT group were higher risk for SSI due to significantly older age, higher incidence, diabetes, more levels of instrumented fusion, and EBL, the latter two being likely markers of increased surgical complexity. The present study also includes high-risk patients with infection, tumor, and deformity, who were notably excluded from some prior studies [4, 13]. In particular, the ciNPWT group had significantly more surgeries for deformity than controls. Additionally, surgeries involved long constructs with an average of 5 intervertebral levels instrumented across for all patients. Despite these predisposing factors placing patients at high risk for infection, the overall reported infection rate in the present study (13.1%) does not differ substantially from previously reported studies involving posterior instrumentation by Adogwa et al. (13.67%) and Mehkri et al. (14.42%) [4, 12]. Overall incidence of infection in the present study is high, and therefore the attributed effect of ciNPWT may not be applicable to practices with lower risk patients/surgeries. The use of hospital costs as an outcome is complicated by significant variations in costs across time, region, and hospital system. Its importance, however, cannot be understated given the significant financial and healthcare resource investment associated with both the index spine surgery as well as management of wound dehiscence and SSI. Future studies should evaluate which patient populations and surgical modifiers may receive the greatest from application of an ciNPWT.

## Conclusion

Prophylactic ciNPWT significantly reduced the incidence of wound dehiscence and SSI by 36.9% and 60.7%, respectively. Additionally, ciNPWT represents a relatively low-cost intervention which portends a mean SSI-associated costs savings of $270,775 per 100 patients receiving posterior instrumented spinal fusion.

## Electronic supplementary material

Below is the link to the electronic supplementary material.

Supplementary Material 1

## Data Availability

No datasets were generated or analysed during the current study.
