# Supplementary material for: Prophylactic closed-incisional negative pressure wound therapy following posterior instrumented spinal fusion: a single surgeon’s experience and cost-benefit analysis
Source: Neurosurg Rev. 2024 Nov 15;47(1):847. doi: 10.1007/s10143-024-03083-8 (PMC11564409; doi:10.1007/s10143-024-03083-8)
Supplement: Supplementary file 1 — Supplementary Material 1 [file 10143_2024_3083_MOESM1_ESM.docx]

**Cost-Analysis Data**

Average cost of ciNPWT - $570.08 (rounded down to $570)

Average cost of SSI treatment - $26,222.36 (rounded down to $26,222)

NNT to prevent one SSI – 7.79 (rounded up to 8)

Cost to prevent 1 SSI = Cost of ciNPWT (rounded) * NNT

- Cost to prevent 1 SSI = 570 * 8
  - Cost to prevent 1 SSI = $4,560

Mean cost savings per NNT = Cost of SSI treatment – Cost to prevent 1 SSI

- Mean cost savings per NNT = 26222 – 4560
  - Mean cost savings per NNT = $21,662

Mean cost savings per 100 surgeries = Mean cost savings per NNT / 8 * 100

- Mean cost savings per 100 surgeries = 21662 / 8 * 100
  - Mean cost savings per 100 surgeries = $270,775
